# Supplementation with Fish Oil Improves Meat Fatty Acid Profile although Impairs Growth Performance of Early Weaned Rabbits

**DOI:** 10.3390/ani9070437

**Published:** 2019-07-11

**Authors:** María Rodríguez, María Dolores Carro, Víctor Valiente, Nora Formoso-Rafferty, Pilar G. Rebollar

**Affiliations:** 1 Departamento de Producción Agraria, Escuela Técnica Superior de Ingeniería Agronómica Alimentaria y de Biosistemas, Universidad Politécnica de Madrid, Ciudad Universitaria s/n, 28040 Madrid, Spain; 2 Departamento de Producción Animal, Facultad de Veterinaria, Universidad Complutense de Madrid, Ciudad Universitaria, s/n, 28040 Madrid, Spain

**Keywords:** rabbit, early weaning, fish oil, carcass quality, polyunsaturated fatty acids, fatty acid profile

## Abstract

**Simple Summary:**

Early weaning is a practice commonly applied in rabbit production despite its possible detrimental consequences on production, as it negatively affects the welfare of young rabbits that are highly susceptible to digestive disturbances. The inclusion of long-chain n-3 polyunsaturated fatty acids (PUFA) in the mother’s diet during the suckling period can bring substantial benefits to the kits, as PUFA are involved in the development of the immune response in early weaned animals and could reduce morbidity and mortality during the fattening period. In this study, we observed that including fish oil (containing PUFA) in the diet of early weaned rabbits can reduce their morbidity and enhance the content of beneficial long-chain n-3 fatty acids in rabbit meat and fat, but it also slightly reduces their growth.

**Abstract:**

Our objective was to analyze the influence of replacing lard (control) with fish oil (FO) rich in long-chain n-3 polyunsaturated fatty acids in the diet of rabbits weaned at 25 days of age on their growth performance, meat quality, cecal fermentation, and ileal morphology. Twenty-four litters (12 control and 12 FO) of nine kits each were fed the experimental diets from weaning (25 days) until slaughter at 60 days of age. Half of the litters (six per diet) were used to monitor productive performance, carcass characteristics, and ileal morphology, and cecal fermentation was assessed in the rest of litters. Diet had no influence on feed intake, meat color, and pH or cecal fermentation, but FO-fed rabbits had lower (*p* ≤ 0.049) average daily gain, chilled carcass weight, and perirenal fat than control rabbits. Fish oil inclusion in the diet resulted in lower morbidity (5.56% vs. 20.4%; *p* = 0.019) and a healthier fatty acid profile with lower (*p* < 0.001) n-6/n-3 ratios in both muscle and perirenal fat. In summary, the inclusion of FO in the diet of early weaned rabbits improved the fatty acid profile of rabbit meat and fat and decreased the morbidity, but growth performance was slightly reduced.

## 1. Introduction

Rabbit meat is a Mediterranean food that is considered to be especially appreciated by consumers for its low lipid and cholesterol content and the high biological value of its protein [1,2]. However, despite being a meat that offers excellent nutritive and dietetic properties, in practical dietary conditions, their n-6/n-3 polyunsaturated fatty acids (PUFA) ratio is frequently above the optimal values recommended for human consumption [3,4,5]. Usually, the most common sources of fat in feed formulation of fattening rabbits are tallow, lard, deodorized oleins, and sunflower oil [6], which are low in n-3 PUFA. The manipulation of the diet of rabbits has proved to be very effective in producing PUFA-enriched meat, making it relatively easy to achieve optimal values [4,7]. Fish oil (FO) is one of the most common animal sources of n-3 PUFA, as it contains higher PUFA amounts than seed oils [8].

Weaning is a stressful period for rabbits because of the nutritional transition from milk to solid feed and the social events related to new housing and litter management. A recent study [9] using PUFA-enriched diets during pregnancy and lactation periods corroborated the presence of long-chain PUFA in rabbit milk, which combined with other components could help to prevent digestive disturbances during the suckling period [10]. Long-chain n-3 PUFA are implicated in the development of the animal immune response [11], and medium-chain fatty acids such as caprylic and capric can alter the intestinal microbiota, thus affecting cecal fermentation [12] and the development of pathogenic strains [13]. In fact, Maertens et al. [14] observed that in an experimental farm naturally affected by epizootic rabbit enteropathy, the mortality of animals weaned at 32 days of age was reduced by including extruded linseed and decreasing the dietary n-6/n-3 ratio in the mother’s diet. Nonetheless, in a previous study by our group [4] feeding PUFA-enriched diets to rabbits weaned at the same age (32 days), no effects were observed regarding mortality.

On the other hand, several studies have demonstrated the possibility of successfully anticipating kit weaning age from 32 to 25 days of age [15,16,17]. The major interest of this practice is the possibility of reducing the doe energy deficit by shortening the lactation length and extending the dry period, thus helping the does to recover the body reserves mobilized during the lactation [18], which could increase their productive life. However, this strategy can negatively affect the growth and the mortality rates of kits because solid feed intake is increased, modulating the digestive tract maturation and resulting in a higher concentration of volatile fatty acids (VFA) and a lower pH in the cecum [19]. In addition, the early withdrawal of milk has an indirect negative effect on healthy kits that have higher sensitivity to digestive disturbances [20,21].

Our hypothesis was that fish oil supplementation to early weaned rabbits would positively affect animal performance by reducing morbidity and/or mortality and improving meat FA profile. Therefore, the objective of this study was to analyze the influence of replacing lard with fish oil in the diet of rabbits weaned at 25 days of age on growth performance, meat quality, cecal fermentation, and ileal morphology.

## 2. Materials and Methods

### 2.1. Diets, Animals, and Experimental Design

The two experimental diets had the same feed ingredients and only differed in the type of fat, which was either 0.75% lard (control) or 1.5% Optomega-50 (FO), a commercial supplement based on salmon oil (Optivite International Ltd., Barcelona, Spain) and containing 50% of ether extract (35% of n-3 PUFA, 8% of n-6 PUFA, and 2500 mg/kg of vitamin E) on a mineral-based carrier. Sepiolite was included at 0.75% in the control diet to equal the carrier of Optomega-50. As the diets contained the same feeds and amount of fat, they were isoenergetic and isoproteic. The ingredient and chemical composition of the diets have been reported by Rodríguez et al. [4]. Both diets contained wheat bran, barley grain, sunflower meal, palm kernel, lucerne meal, barely sprouts, sugar beet pulp, sugarcane molasses, wheat straw, and additives in proportions of 30.0, 11.1, 19.9, 6.0, 10.0, 5.0, 5.7, 3.0, 4.2, and 3.6 g/100 g diet (fresh matter basis), respectively. The control diet contained (on a dry matter (DM) basis excepting gross energy and DM) 16.4 MJ/kg, 90.6% DM, 8.11% ash, 16.0% crude protein, 3.16% ether extract, and 33.2% neutral detergent fiber, respectively, whereas the FO diet contained 16.5 MJ/kg, 90.4% DM, 7.88% ash, 16.0% crude protein, 3.14% ether extract, and 31.6% neutral detergent fiber, respectively. The FA profile of the two experimental diets is given in Table 1.

All procedures involving animals were approved by the Animal Ethics Committee of the Community of Madrid (license number PROEX 302/15) and were in compliance with the Spanish Guidelines for Care and Use of Animals in Research [22]. Stable environmental conditions (19–22 °C; 15 air renovations per h; 50% ± 5% humidity; 16 h of light and 8 h of darkness) were maintained throughout the trial.

Twenty-four rabbit does (12 control and 12 FO does) housed individually with their corresponding litters (of 10–11 kits) were fed the experimental diets during pregnancy and lactation. All kits were weaned after 25 days of lactation, and each litter was housed in an independent flat-deck cage (60 × 50 × 33.5 cm). All kits of each litter were weighed and subsequently standardized to nine kits per cage (kits with a similar body weight to the average litter value), removing the outliers. Rabbits continued to be fed their maternal diet ad libitum until slaughter at 60 days of age. Viability of rabbits was monitored twice a day (at 8:00 a.m. and 8:00 p.m.) throughout the entire trial. To avoid possible erroneous determinations of feed intake in each cage, rabbits with signs of low viability (lethargy/weakness, crouched posture, rough coat) were substituted by others which had been fed the same experimental diet and housed at the beginning of the trial in separate cages under the same conditions. Half of the litters (six per each diet) were used to monitor productive performance, carcass characteristics, and ileal morphology. In the other half, cecal fermentation was assessed.

### 2.2. Productive Performance, Carcass Characteristics, and Ileal Morphology

The initial average body weight of kits was 332 ± 27.0 and 328 ± 22.1 g for control and FO groups, respectively. All rabbits were weighed weekly and feed intake was monitored every seven days to determine the feed conversion ratio. At day 60 days of age, all rabbits were weighed and four animals from each litter, having a similar body weight to that of the average litter value, were euthanized. Firstly, rabbits were stunned at low voltage (90 V; 5 s) and then were bled by cutting the carotid arteries and jugular veins. The skin, full gastrointestinal tract, urogenital tract, distal legs, and the tail were removed, and the carcasses were weighed. The perirenal, suprascapular, and abdominal fat of the slaughtered animals were removed and weighed individually, and samples (2–3 g) of both perirenal fat and *Longissimus lumborum* were taken for analysis of FA profile and immediately frozen at −20 °C. The pH of the *Longissimus lumborum* was measured on the seventh lumbar vertebrae using a portable Crisson 25 pHmeter with a penetration electrode 5053 T (Crisson Instruments, Barcelona, Spain) at 0 min, 30 min, and 24 h postmortem. Meat color was assessed in the *Longissimus lumborum* by the L *a *b * system [23] using a Konica Minolta CM-700d colorimeter (Azuchi-Machi Higashi-Ku, Osaka 541, Japan). The carcasses were then hanged and maintained for 24 h in a ventilated cold room (3–5 °C) before being weighed to calculate the drip loss percentage and cold carcass yield. Finally, the left hind leg was separated and dissected to determine the amount of meat, bone, and fat. All the procedures described previously were in accordance with the recommendations of the World Rabbit Science Association described by Blasco et al. [24].

For ileal morphology analyses, a 4 cm sample of ileum was taken next to the last Peyer’s patch from two rabbits of each litter. Samples were processed as described by Rodríguez et al. [4] before cutting histological sections of 5 µm, which were analyzed by light microscopy (Olympus BX40; Olympus Optical Co., Hamburg, Germany) using the Soft Imaging System (Olympus, GmbH, Hamburg, Germany). Villus length and crypt depth were measured according to Hampson [25] and the villus length/crypt depth ratio was calculated in four cross sections from the mean value of 30 vertically oriented villi per rabbit.

### 2.3. Cecal Fermentation

Cecal fermentation was assessed in the rest of litters (six per diet, each having nine rabbits), which were maintained under the same housing and feeding conditions described before. The initial average body weight of the kits was 331 ± 10.6 and 329 ± 11.0 g for control and FO groups, respectively. Two rabbits with similar body weights to the average litter value were selected in each litter at 25, 45, and 60 days of age and slaughtered by cervical dislocation. The cecum was immediately removed and weighed. The content was extracted, weighed, and homogenized, and the pH was measured (Crison Basic 20 pHmeter; Crison Instruments, Barcelona, Spain). Cecal content was sampled, 2 g were mixed with 2 mL of 0.5 *N* HCl, and samples were immediately frozen (−20 °C) until analysis of VFA. The rest of cecal content was used to determine DM content.

### 2.4. Chemical Analyses

Chemical analysis of experimental diets followed the official methods of the AOAC [26] for DM (ID 934.01), ash (ID 923.03), and ether extract (ID 920.39). Nitrogen content was analyzed by the Dumas method (ID 968.06) using a FP-528 LECO (LECO, St. Joseph, MI, USA) and gross energy was determined by combustion in an adiabatic calorimetric pump (model 1356, Parr Instrument Company, Moline, IL, USA). The neutral detergent fiber, acid detergent fiber, and acid detergent lignin contents were determined according to the sequential method of Van Soest et al. [27] in an ANKOM220 Fiber Analyzer unit (ANKOMTechnology Corporation, Fairport, NY, USA) and using sodium sulfite and heat-stable amylase. Results are expressed exclusive of residual ash.

Lipids from dietary samples were extracted as described by Sukhija and Palmquist [28]. The lipids from *Longissimus lumborum* samples were extracted using the procedure described by Segura and López-Bote [29]. Briefly, 200 mg of lyophilized samples (in triplicate) were homogenized in dichloromethane:methanol (8:2; v/v) with a mixer mill (MM400; Retsch technology, Stuttgart, Germany) and the final biphasic system was separated by centrifugation. Solvent was evaporated under nitrogen stream, lipids were dried by vacuum desiccation, and total lipid content was determined gravimetrically. Fatty acid methyl esters (FAME) were prepared from total lipids by transesterification using a mixture of sodium methylate–methanol and methylated in the presence of sulfuric acid [29]. The FAME were separated using a gas chromatograph (HP 6890 Series GC System; Hewlett Packard Co., Avondale, PA, USA) equipped with a flame ionization detector and an HP-Innowax polyethylene glycol column (30 m × 0.316 mm × 0.25 µm; J&W Scientific/Agilent Technologies, Santa Clara, CA, USA), and nitrogen as a carrier gas. The FA from perirenal fat samples were extracted using a mixture of chloroform/methanol (2:1, v/v), methylated in the presence of sodium methoxide and quantified as described by Cordero et al. [30] using a Hewlett Packard HP-5890 (Avondale, PA, USA) gas chromatograph equipped with a flame ionization detector (HP-Innowax capillary column, 30 m length, 0.32 mm internal diameter, and 0.25 m film thickness; Agilent Technologies Gmbh, Germany). A split ratio of 50:1 was used and C15:0 was included as the internal standard.

For VFA analysis, cecal samples were thawed and centrifuged as described by García-Martínez et al. [31]. The VFA concentration was determined by gas chromatography using a Perkin Elmer Autosystem XL gas chromatograph (Perkin Elmer Inc., Shelton, CT, USA) equipped with an automatic injector, detector flame ionization, and a semicapillary column TR-FFAP 30 m × 0.53 mm × 1 μm (Supelco, Barcelona, Spain) under the conditions described by García-Martínez et al. [31].

### 2.5. Statistical Analyses

The experimental unit for all measured parameters was the cage. Data on feed intake, average daily gain, feed conversion ratio, and cecal fermentation were analyzed as a mixed model with repeated measures using the PROC MIXED of SAS (SAS Inst. Inc., Cary, NC, USA). The statistical model included the experimental diet, time (day of sampling), the interaction between diet and time as fixed effects, and the cage as a random effect. The rest of the data were analyzed by the same model excluding the effects of time. Significance was declared at *p* < 0.05, and *p*-values between 0.05 and 0.10 were considered as trends. Morbidity data in the growth performance trial were analyzed using the PROC CATMOD of SAS. All results are presented as least-squares means.

## 3. Results

### 3.1. Growth Performance, Carcass Characteristics, and Ileal Morphology

Morbidity during experimental period was lower (*p* = 0.019) in the FO-fed group (5.56%) than in the control one (20.4%). As shown in Table 2, there were no differences between groups in feed intake, but FO-fed rabbits had a lower (*p* = 0.049) average daily gain and tended (*p* = 0.083) to show greater feed conversion ratios compared with the control group. No diet and time interactions were detected for any of these parameters.

At slaughter, rabbits from FO group tended to have a lower body weight and hot carcass weight (*p* = 0.080 and 0.058, respectively) and had a lower (*p* = 0.036) chilled carcass weight than control rabbits (Table 3). The type of fat in the diet did not affect any color parameter or the pH of the meat measured at 0 min, 30 min, and 24 h after slaughter. Replacing lard with fish oil did not result in changes in the drip loss percentage, but FO-fed rabbits tended to have lower weights of skin and full gastrointestinal tract (*p* = 0.090 and 0.089, respectively). Moreover, FO-fed rabbits had a lower (*p* = 0.003) amount of perirenal fat and tended (*p* = 0.066) to have a lower amount of scapular fat compared with those fed the control diet. Diet did not affect ileal villus length and villus length/crypt depth ratio, but crypt depth was greater (*p* = 0.012) in control rabbits than in those fed the FO diet (Table 3).

The weight of the left hind leg tended to be lower (*p* = 0.079) and its bone proportion tended to be higher (*p* = 0.083) in FO-fed rabbits than in the control ones, but no differences were detected in the proportion of muscle and fat in the left hind leg (Table 3).

There were no differences between groups in moisture and total fat content in the *Longissimus lumborum*. Table 4 and Table 5 show the FA profile of *Longissimus lumborum* and perirenal fat, respectively. Replacing lard with fish oil tended to reduce (*p* = 0.058) total PUFA concentrations in the *Longissimus lumborum* but increased (*p* = 0.011) them in the perirenal fat. The C20:5n-3, C22:5n-3, and C22:6n-3 content of *Longissimus lumborum* was 1.8, 1.4, and 3.6 times greater (*p* < 0.001–0.018) in the FO-fed rabbits than in the control ones. In addition, FO-fed rabbits showed greater concentrations of C18:3n-3 in both the *Longissimus lumborum* (*p* = 0.008) and perirenal fat (*p* < 0.001) than control rabbits.

Total monounsaturated fatty acids in the perirenal fat of FO-fed rabbits tended to be lower (*p* = 0.084) than in control ones, but there were no differences due to the diet in the *Longissimus lumborum*. Moreover, C20:5n-3 and C22:6n-3 were not detected in the perirenal fat of control rabbits, but both fatty acids were detected in the *Longissimus lumborum* of the same animals. The n-6/n-3 ratio in the *Longissimus lumborum* of FO-fed rabbits was in the range recommended for healthy eating, whereas meat from control rabbits slightly exceeded this value (1.73 vs. 4.32, respectively; *p* < 0.001).

### 3.2. Cecal Fermentation

There were no differences (*p* = 0.171) between groups in the body weight of the rabbits slaughtered at 25, 45, and 60 days of age for cecal sampling, and no diet and time interaction was observed (*p* = 0.826) for body weight (results not shown). Diet had no influence on the weight of full cecum (*p* = 0.111) and cecal contents (*p* = 0.151) at any sampling time (averaged values across sampling times: 64.3 and 43.2 g for control diet rabbits, and 60.6 and 40.6 g for FO diet rabbits, respectively; data not shown). There were no diet and time interactions (*p* = 0.287–0.851) for any analyzed parameter. As shown in Table 6, fish oil supplementation did not affect cecal dry matter content, pH values, and total VFA concentrations. There were no differences between groups in VFA profile, with the exception that the propionate proportion was lower (*p* = 0.014) in the FO group compared with control rabbits. Total VFA concentrations, dry matter content, and molar proportions of acetate and propionate in the cecum increased (*p* < 0.001, 0.023, and 0.001, respectively), whereas pH values and butyrate proportions values decreased (*p* < 0.001) from 25 to 60 days of age.

## 4. Discussion

### 4.1. Growth Performance, Carcass Characteristics, and Ileal Morphology

The use of a supplement based on n-3 PUFA from fish oil during the fattening period did not improve the productive parameters of rabbits but modified the quality of their carcass. Moreover, the greater risk of disease due to the early weaning applied in this study could be alleviated by PUFA’s positive effect on the immune system [32]. It seems the effects of omega-3 fatty acids may be reducing the effect of inflammation and, consequently, a reduced morbidity during the experimental period was observed. Similar results were found in broilers challenged with simulated disease [33,34] and piglet vitality by Tanghe and De Smet [35]. The inclusion of FO in animal diets results in a decrease in the amount of arachidonic acid and an increase of n-3 PUFA incorporated into membrane phospholipids of cells involved in inflammation, reducing leukocyte chemotaxis and modifying production of inflammation mediators or eicosanoids such as prostaglandins, thromboxanes, lipoxins, and leukotrienes (reviewed by Calder [36]). Due to their mothers also being fed the same diets during pregnancy and lactation, it is possible that there was a metabolic programming in these young animals, which was observed in a previous study [37] where a favorable hyperlipidemic status of neonates with positive implications for their survival was detected.

Although there were no differences between groups in feed intake, FO-fed rabbits had lower daily gain, hot and chilled carcass weight, and tended to show greater feed conversion ratios compared with control rabbits. These findings contrast with previous results of our group [4], in which the same experimental diets were fed to 30-day weaned rabbits and no effects of fish oil supplementation were observed either on daily gain, carcass traits, or feed conversion ratio. Early weaning usually provokes impairment in the nutrient caption by enterocytes [38], but PUFA supplementation can improve the development of some tissues and systems [39,40]. The effects of isocaloric modifications in the PUFA/saturated fatty acids ratio of diets in relation to the intestinal uptake of nutrients reported in the literature are controversial. For instance, glucose uptake in the intestine is downregulated by feeding a high PUFA/saturated fatty acid diet according to Thomson et al. [41], but Gabler et al. [42] observed an increase in glucose uptake after the incorporation of n-3 PUFA in the diet. In our study, we might speculate that FO reduced nutrient absorption in rabbits, resulting in lower daily gain. Nonetheless, although FO-fed rabbits had lower ileal crypt lengths, the lack of differences between groups in the villus length/crypt depth ratio, which is regarded as a general indicator of intestinal functional state [43], would indicate no damage of the ileal epithelium in FO-fed rabbits. Other supplements used in rabbit diets during the fattening period [44,45] have also reported lower daily gains in rabbits without differences in feed intake. Attia et al. [44] observed that the inclusion of inulin (3.7%) as an alternative to antibiotics in the postweaning period significantly decreased the daily gain between 57 and 81 days of age without affecting feed intake and diet digestibility. Inulin and other mannan-oligosaccharides can influence rabbit growth, but their effects on nutrient digestibility are variable [46]. Besides, as is known, differences in housing conditions can also influence both feed intake and daily gains. For instance, Loponte et al. [45] observed that free-range rabbits had a higher feed intake than cage-housed rabbits, but their daily gain was lower, which was attributed to the increase in the energy requirements of free-range rabbits due to their higher activity; however, in our study, housing conditions were the same for both groups.

The effects of PUFA supplementation on feed intake and growth performance reported in the literature are contrasting. Kowalska and Bielanski [47] observed no effects of fish oil supplementation (3% diet) on growth performance and feed conversion efficiency of rabbits. Similarly, Trebušak et al. [48] detected no changes in rabbit growth when the diet was supplemented with PUFA-rich fats (palm fat with 99% saturated fatty acids vs. linseed oil with more than 70% PUFA). In contrast, others [49,50] have observed reduced growing performance when linseed or linseed oil was included in the diet. It seems that both the fatty acid profile of the supplemented fat and the level of PUFA supplementation can be involved in the variable responses observed in the different studies. Feed intake, average daily gain, and feed conversion ratio values in the present study were similar to those reported previously for similar production conditions [47,51,52]. As reported by others [53,54], feed conversion ratio increased with time due to the better feed efficiency in young animals than in older ones [55].

In agreement with previous results [4,56], the type of fat in the diet did not affect any color parameter or the pH of the meat. The lower contents of perirenal and scapular fat, both of which are usually included in the rabbit carcass, observed in FO-fed rabbits in our study are in accordance with previous studies [4,47,57,58], reporting a decrease in carcass fat content after PUFA-rich fat supplementation. Dietary supplementation of n-3 PUFA has resulted in a reduction of lipogenesis in different animal species [59,60], and it has been linked to the stimulation of hepatic fatty acid oxidation [61]. The trend to lower skin weight observed for FO-fed rabbits agrees with previous results by our group [4], and it has been attributed to a higher deposition of subcutaneous fat with a more saturated profile in control rabbits; this would result in higher fat densification, making subcutaneous fat more easily removed at skinning in the control than in the FO group. The similar tissue proportions of muscle and fat in the left hind leg were like those previously reported by others in rabbits when fish oil [45] or other PUFA-rich fats [7,56,62] were included in the diet.

As expected, C20:5n-3, C22:5n-3, and C22:6n-3 were present in the *Longissimus lumborum* of FO-fed rabbits, but these fatty acids were also detected in the *Longissimus lumborum* of all control rabbits, which indicates the ability of rabbits to synthesize endogenous n-3 fatty acids [63]. In agreement with our results, others [63,64,65] have reported an increase in the C20:5n-3, C22:5n-3, and C22:6n-3 content in rabbit carcasses after supplementing the diet with C18:3n-3. The level at which C18:3n-3 can be converted to C20:5n-3 and C22:6n-3 seems to vary with the body tissue, and the heart and the liver have been reported to have a greater capacity compared with the skeletal muscle [63]. In accordance with our results, many studies have reported the marked influence of the dietary fatty acid profile on fatty acid deposition in rabbit carcasses [63,64,65,66,67]. In addition, the n-6/n-3 ratio in the *Longissimus lumborum* of FO-supplemented rabbits was in the range (<4.0) recommended for healthy eating [68], whereas meat from control rabbits slightly exceeded this value. These results show that meat from PUFA rabbits had higher nutritional quality than that from control rabbits.

### 4.2. Cecal Fermentation

The lack of differences between groups on cecal fermentation parameters in our study is in contrast with the results of Rodríguez et al. [4], who observed that rabbits weaned at 30 days of age and receiving the same FO diet used in the present study showed significantly lower DM content and greater VFA concentrations in the cecum than control rabbits and attributed the differences to increased microbial activity in control animals. The reason for the different response observed is unknown, but it might be related to differences in the intestinal microbiota of the rabbits in both experiments. Other studies have reported that some fatty acids may have antimicrobial activity on selected microorganisms [12,13,69], but to the best of our knowledge, there is no information on the specific effects of fish oil fatty acids on gut microbiota in rabbits, although an active anaerobe microbial lipid metabolism has been demonstrated [70]. In rats, fish oil supplementation has resulted in lower pH of cecal digesta and greater butyrate concentrations [71]. The reason for the reduced propionate proportions in the cecum of the FO-fed rabbits is unknown, but it might reflect changes in cecal microbiota caused by increased n-3 PUFA content in the milk, as rabbit does received the same experimental diet as their litters during gestation and lactation. In fact, increased PUFA concentrations in the milk of does supplemented with fish oil has been demonstrated in a previous study by our group [9]. Variations in total VFA concentration and pH values over the fattening period, which increased and decreased with advanced age, respectively, were in accordance with results from previous studies [4,72].

## 5. Conclusions

Replacing lard with fish oil in the diet of early weaned rabbits reduced the morbidity and changed the fatty profile of perirenal fat and meat to a more favorable profile for human nutrition. However, the inclusion of fish oil in the diet slightly reduced the growth performance of the rabbits.

## Figures and Tables

**Table 1:** Fatty acid composition (g/100 g of total fatty acid methyl esters) of diets with either lard (control) or with fish oil (FO) as the fat source.

Fatty Acid	Diet	
	Control	FO
C12:0	6.33	6.36
C14:0	5.32	6.10
C16:0	18.3	16.62
C18:0	5.42	2.76
Total saturated fatty acids	35.4	31.8
C16:1n-7	1.34	1.68
C18:1n-9	24.1	17.7
C18:1n-7	1.59	1.23
C20:1n-9	1.31	1.21
Total monounsaturated fatty acids	28.3	21.8
C18:2n-6	32.7	31.5
C18:3n-3	4.08	4.45
C18:4 n-3	0.51	2.16
C20:5n-3	ND ^1^	3.39
C22:5n-3	ND ^1^	0.92
C22:6n-3	ND ^1^	4.00
Total polyunsaturated fatty acids PUFA	36.6	46.4
n-9	25.4	18.9
n-6	33.5	32.8
n-3	4.59	14.9
n-6/n-3 ratio	7.29	2.20

^1^ ND = not detected.

**Table 2:** Feed intake, daily gain, and feed conversion ratio of fattening rabbits fed diets with either lard (control) or fish oil (FO) as the fat source from 25 to 60 days of age ^1^.

Item	Week of Growing Period					Average	SEM_D_^2^	SEM_T_^2^	*p*-Value		
	1	2	3	4	5				Diet	Time	Diet × Time
Average daily feed intake (g/day)											
Control	49.9	103	110	126	144	107	2.32	2.84	0.914	<0.001	0.739
FO	50.8	105	116	121	140	106					
Average daily gain (g/day)											
Control	34.2	41.6	49.1	50.7	49.8	45.0	0.87	1.14	0.049	<0.001	0.879
FO	30.5	38.4	46.6	49.5	45.6	42.1					
Feed conversion ratio (g/g)											
Control	1.44	2.56	2.28	2.49	2.91	2.34	0.061	0.074	0.083	<0.001	0.687
FO	1.68	2.72	2.49	2.45	3.07	2.48					

^1^ Six litters of nine kits each were fed each diet. All values are least-squares means. ^2^ SEM_D_ and SEM_T_: standard error of the mean for diet and time effects, respectively.

**Table 3:** Live body weight at slaughter, carcass traits, tissue composition of the left hind leg, and ileal morphology of growing rabbits fed diets with either lard (control) or fish oil (FO) as the fat source and slaughtered at 60 days of age ^1^.

Item	Diet		SEM	*p*-Value
	Control	FO		
Body weight (g)	1968	1861	32.1	0.080
Carcass traits				
Hot carcass weight (g)	1193	1112	20.9	0.058
Hot carcass yield (%)	60.6	59.7	0.31	0.082
Chilled carcass weight (g)	1158	1052	20.7	0.036
pH				
Slaughter time	7.51	7.44	0.044	0.332
30 min	7.00	7.02	0.063	0.745
24 h	6.06	6.03	0.048	0.684
Color				
L * ^2^	51.9	52.9	0.580	0.359
a * ^3^	7.56	7.46	0.522	0.898
b * ^4^	15.0	14.2	0.361	0.530
Drip loss percentage (%)	3.72	5.59	0.537	0.176
Skin weight (g)	355	336	7.78	0.090
Full gastrointestinal tract weight (g)	361	340	6.38	0.089
Fat (g)				
Abdominal	24.7	25.2	0.80	0.767
Scapular	7.40	6.14	0.481	0.066
Perirenal	13.7	11.2	0.48	0.003
Left hind leg (g)				
Total weight (g)	160	147	3.0	0.079
Proportion of (g/100 g):				
Bone	15.8	16.5	0.218	0.083
Muscle	73.9	74.2	0.992	0.880
Fat	5.34	5.41	0.292	0.886
Ileal morphology				
Crypt depth (µm)	142	131	3.2	0.012
Villus length (µm)	585	550	16.6	0.169
Villus length/crypt depth	4.12	4.20	0.102	0.386

^1^ 24 rabbits per group. All values are least-squares means. ^2^ L*: lightness from black (0) to white (100). ^3^ a*: from green (−) to red (+). ^4^ b*: from blue (−) to yellow (+).

**Table 4:** Total lipid content and fatty acid profiles in the *Longissimus lumborum* muscle of growing rabbits fed diets with either lard (control) or fish oil (FO) as the fat source and slaughtered at 60 days of age ^1^.

Item	Diet		SEM	*p*-Value
	Control	FO		
Total lipids (g/100 g)	1.63	1.52	0.034	0.439
Fatty acid profile (g/100 g of total methyl esters)				
C12:0	0.32	0.44	0.028	0.022
C14:0	2.05	2.69	0.121	0.017
C16:0	21.6	22.9	0.322	0.013
C18:0	6.53	6.48	0.093	0.892
C20:0	0.15	0.17	0.031	0.636
Total saturated fatty acids	37.9	39.5	0.205	<0.001
C16:1n-7	2.41	2.62	0.145	0.463
C18:1n-9	19.0	18.9	0.284	0.877
C18:1n-7	1.18	1.30	0.033	0.025
Total monounsaturated fatty acids	24.7	24.9	0.403	0.784
C18:2n-6	20.4	16.0	0.226	<0.001
C18:3n-3	0.75	1.01	0.048	0.008
C20:3n-9	0.73	0.59	0.022	0.002
C20:4n-6	4.94	4.07	0.132	0.019
C20:5n-3	0.85	1.57	0.069	<0.001
C22:5n-3	3.30	4.67	0.193	0.018
C22:6n-3	1.28	4.58	0.107	<0.001
Total polyunsaturated fatty acids	37.4	36.0	0.437	0.058
n-6	25.4	20.1	0.272	<0.001
n-3	6.17	11.7	0.241	<0.001
n-6/n-3 ratio	4.32	1.73	0.198	< 0.001

^1^ 12 rabbits per group. All values are least-squares means.

**Table 5:** Fatty acid profiles (g/100 g total fatty acid methyl esters) of perirenal fat of growing rabbits fed diets with either lard (control) or fish oil (FO) as the fat source and slaughtered at 60 days of age ^1^.

Fatty Acid	Diet		SEM	*p*-Value
	Control	FO		
C12:0	1.19	1.46	0.021	0.006
C14:0	4.43	4.86	0.037	<0.001
16:0	28.8	27.6	0.304	0.016
C18:0	5.06	4.84	0.084	0.190
C20:0	0.11	0.10	0.002	0.017
Total saturated fatty acids	40.9	40.3	0.297	0.203
C16:1n-7	4.09	4.03	0.109	0.851
C18:1n-9	26.0	23.9	0.196	0.001
C18:1n-7	1.34	1.61	0.051	0.018
Total monounsaturated fatty acids	32.7	31.5	0.292	0.084
C18:2n-6	23.9	22.3	0.356	0.009
C18:3n-3	1.79	2.50	0.035	<0.001
C20:3n-9	0.05	0.08	0.007	0.038
C20:4n-6	0.15	0.18	0.006	0.001
C20:5n-3	ND^2^	0.67	0.018	<0.001
C22:5n-3	0.06	0.76	0.016	<0.001
C22:6n-3	ND^2^	1.28	0.022	<0.001
Total polyunsaturated fatty acids	26.14	27.9	0.409	0.011
n-6	24.3	22.7	0.365	0.014
n-3	1.85	5.20	0.069	<0.001
n-6/n-3 ratio	13.2	4.38	0.199	<0.001

^1^ 12 rabbits per group. All values are least-squares means. ^2^ Not detected.

**Table 6:** Values of pH, dry matter content, and volatile fatty acid (VFA) concentrations in the cecum of growing rabbits fed diets with either lard (control) or fish oil (FO) as the fat source and slaughtered at 25 (weaning), 45, and 60 days of age ^1^.

Item	Age (d)			SEM_D_^2^	SEM_T_^2^	*p*-Value		
	25	45	60			Diet	Time	Diet × Time
pH								
Control	6.38	6.07	5.91	0.061	0.075	0.963	<0.001	0.614
FO	6.31	6.05	5.99					
Dry matter (%)								
Control	21.4	22.4	22.8	0.90	1.10	0.510	0.023	0.287
FO	20.6	22.4	25.5					
Total VFA (mmol/g)								
Control	40.9	50.8	67.4	2.29	2.80	0.510	<0.001	0.667
FO	43.2	54.5	66.0					
Molar proportions, mol/100 mol								
Acetate								
Control	84.6	84.8	78.5	0.58	0.71	0.704	<0.001	0.492
FO	84.1	85.9	78.3					
Propionate								
Control	7.78	4.86	5.02	0.329	0.403	0.014	<0.001	0.503
FO	7.07	3.47	4.56					
Butyrate								
Control	6.96	9.92	15.9	0.494	0.605	0.247	<0.001	0.851
FO	7.96	10.3	16.02					
Minor VFA ^3^								
Control	0.59	0.43	0.58	0.149	0.182	0.820	0.102	0.435
FO	0.89	0.25	0.56					

^1^ Each diet was fed to six litters of nine rabbits each, and two rabbits per litter were slaughtered and sampled at each time (*n* = 12). All values are least-squares means. ^2^ SEM_D_ and SEM_T_: standard error of the mean for diet and time effects, respectively. ^3^ Calculated as the sum of isobutyrate, isovalerate, and valerate.
